# Chronic and reactivated dengue infection in an immunocompromised host: insights from a case report

**DOI:** 10.1186/s41182-025-00779-5

**Published:** 2025-08-14

**Authors:** Ludovic Di Ascia, Etienne Frumence, Nicolas Traversier, Cécile Saint-Pastou, Gilda Grard, Henri Vacher-Coponat, Xavier de Lamballerie, Marie-Christine Jaffar-Bandjee

**Affiliations:** 1 Service de Néphrologie, CHU Réunion Site Nord, Saint-Denis, Réunion France; 2 Laboratoire de Microbiologie, CHU Réunion Site Nord, Saint-Denis, Réunion France; 3 Associated National Reference Center for Arboviruses, CHU Réunion Site Nord, Saint-Denis, Réunion France; 4 Service de Médecine Interne, CHU Réunion Site Sud, Saint-Denis, Réunion France; 5 National Reference Center for Arboviruses, Inserm-IRBA, Marseille, France; 6 UMR Processus Infectieux en Milieu Insulaire Tropical (PIMIT), CNRS 9192, INSERM 1187, IRD 249, Université de La Réunion, Plateforme Technologique CYROI, Sainte-Clotilde, La Réunion France

**Keywords:** Dengue virus, Reactivation, Immunocompromised, Chronic, Case report

## Abstract

**Background:**

Dengue, a viral infection transmitted by mosquitoes, is a growing global health concern, particularly as its spread now puts half of the world’s population at risk. While dengue usually resolves after the primary infection, persistent or chronic cases can occur in immunocompromised individuals.

**Case presentation:**

This case study reports a 43-year-old woman with lupus nephritis and end-stage kidney disease who experienced symptomatic dengue reactivation nearly three years after her initial infection. Despite low viral loads, dengue RNA was detectable in her blood multiple times between 32 and 34 months after the initial detection. Genomic analysis confirmed that the same DENV-1 strain persisted, suggesting chronic infection rather than reinfection. The patient's immunosuppressive treatments, including rituximab, likely impaired her immune response to the initial infection, contributing to viral persistence. Additionally, her profound immunosuppressive state at the time of reactivation, potentially exacerbated by coinfections, may have triggered the virus to re-emerge.

**Conclusion:**

This case highlights the rare but clinically relevant possibility of chronic dengue infection in immunocompromised patients. The confirmed persistence of the same viral strain over nearly three years challenges the conventional view of dengue as a strictly acute infection. It raises concern about the potential for reintroduction and re-emergence of previously circulating strains, as well as the detrimental tissue consequences of chronic infection by the virus. These findings have important implications for clinical management, diagnostic strategies, and public health surveillance, and underscore the need for further research to better understand the mechanisms of dengue chronicity—particularly those involving viral immune evasion and host immune dysfunction.

**Supplementary Information:**

The online version contains supplementary material available at 10.1186/s41182-025-00779-5.

## Background

Dengue is a systemic viral infection caused by the dengue virus (DENV), primarily transmitted by *Aedes aegypti* and *Aedes albopictus* mosquitoes that can cause severe illness in humans and presents significant health, economic, and social challenges [1, 2]. Although typically an acute and self-limiting infection, dengue can, in some cases, progress to severe, life-threatening forms such as dengue hemorrhagic fever, dengue shock syndrome, and dengue organopathies. Currently, dengue is spreading rapidly, driven by the global expansion of *Aedes* mosquitoes and the increase of global travel––involving patients in the viremic phase. These factors have placed half of the world's population at risk, with autochthonous transmission now observed in parts of Europe and the USA [3]. In recent years, dengue has circulated extensively and caused major outbreaks on Reunion Island, a French overseas territory located in the Indian Ocean [4].

There are four distinct serotypes of DENV: DENV-1, DENV-2, DENV-3, and DENV-4. Within days of dengue infection, the levels of DENV-specific antibodies increase in the blood, and innate immune responses and cytotoxic T lymphocytes contribute to the rapid immune clearance of DENV. Primary infection induces the production of type-specific antibodies, which are specific to the infecting serotype and can persist for years [5]. Various subsets of specific T cells against the infecting serotype also prevent symptomatic reinfection by homologous DENV strains. Therefore, DENV is not traditionally considered to persist in the human host. In the general population, DENV detection has been reported to last no more than 11 days in the blood and 1 month in the urine [6].

However, we recently reported cases of persistent dengue detection in urine several months after infection in immunocompromised kidney transplant recipients, with full resolution of DENV infection coinciding with the recovery of CD8 counts [7]. Among these patients, two exhibited a second positive urine test several months after the first, once again coinciding with a decline in total CD8 counts and antibody levels. This strongly suggests that chronic dengue infection is likely facilitated by impaired cellular immunity. Nevertheless, the absence of symptoms during the second detection and the lack of genomic analysis of the strains prevented us from conclusively determining whether this was a case of dengue reactivation.

We report here a case of possible chronic dengue infection in a dialysis patient with an immunocompromised status who presented symptomatic reactivation three years after the initial infection.

## Case presentation

Our case involves a 43-year-old woman who had been followed for 20 years at the University Hospital of Reunion Island for cutaneous articular lupus with anti-DNA and SSA antibodies. Nine years after the initial diagnosis, she developed kidney impairment (class IIIB). Over the course of her treatment, she received multiple lines of immunosuppressive therapies, including corticosteroids, methotrexate, mycophenolate mofetil (MMF), cyclophosphamide, and azathioprine.

**Fig. 1:**
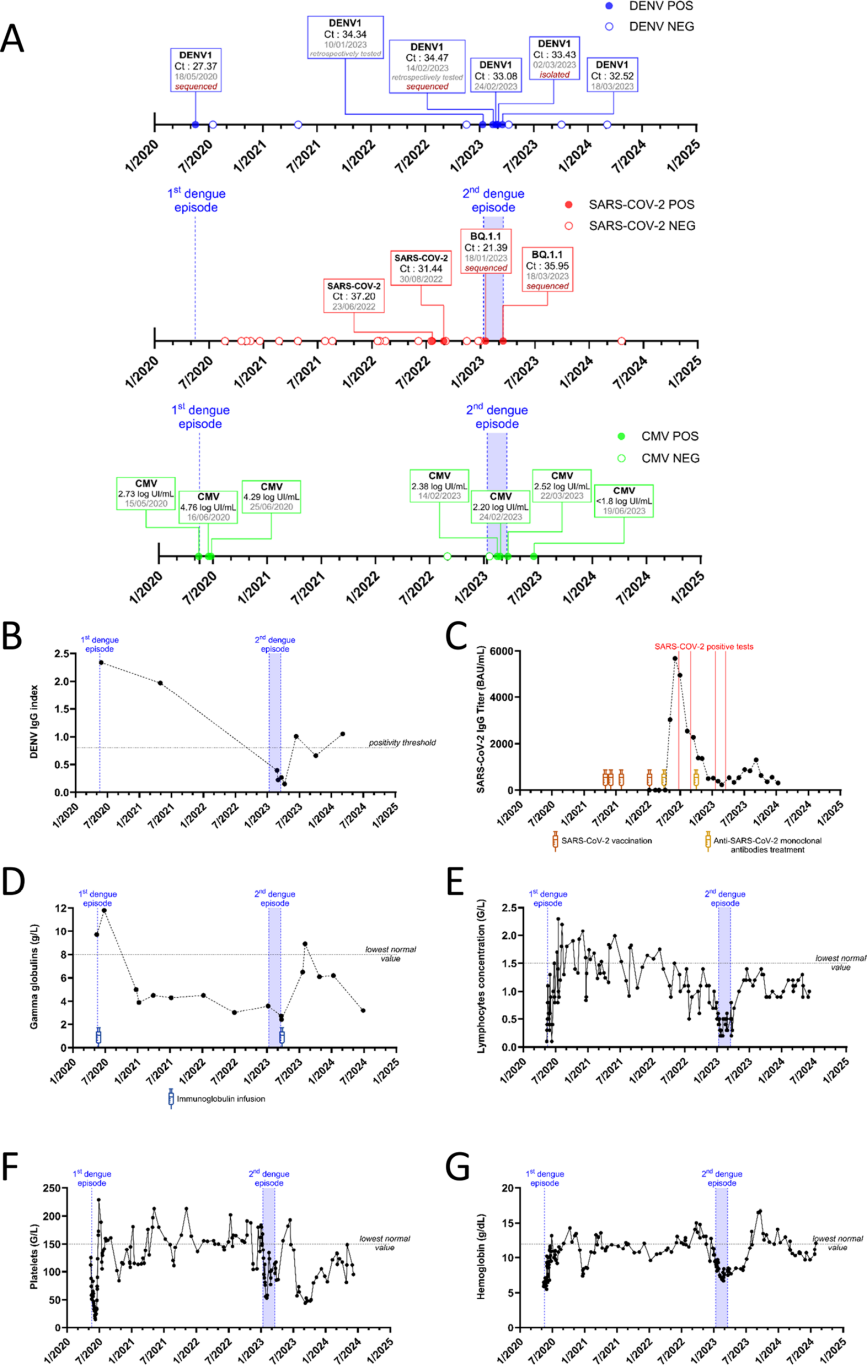
Biological characteristics of the patient from 2020 to 2024. **A** Timeline illustrating the patient’s disease progression, including detection of plasma DENV, nasopharyngeal SARS-CoV-2, and whole-blood CMV by RT-PCR. **B** Levels of anti-DENV IgG measured by ELISA. **C** Levels of anti-SARS-CoV-2 IgG measured by ELISA. **D** Gamma globulin concentrations assessed by protein electrophoresis. **E** Lymphocyte counts measured over this period. (**F**) Platelet concentrations recorded during the same timeframe. **G** Hemoglobin levels assessed throughout this period

**Fig. 2:**
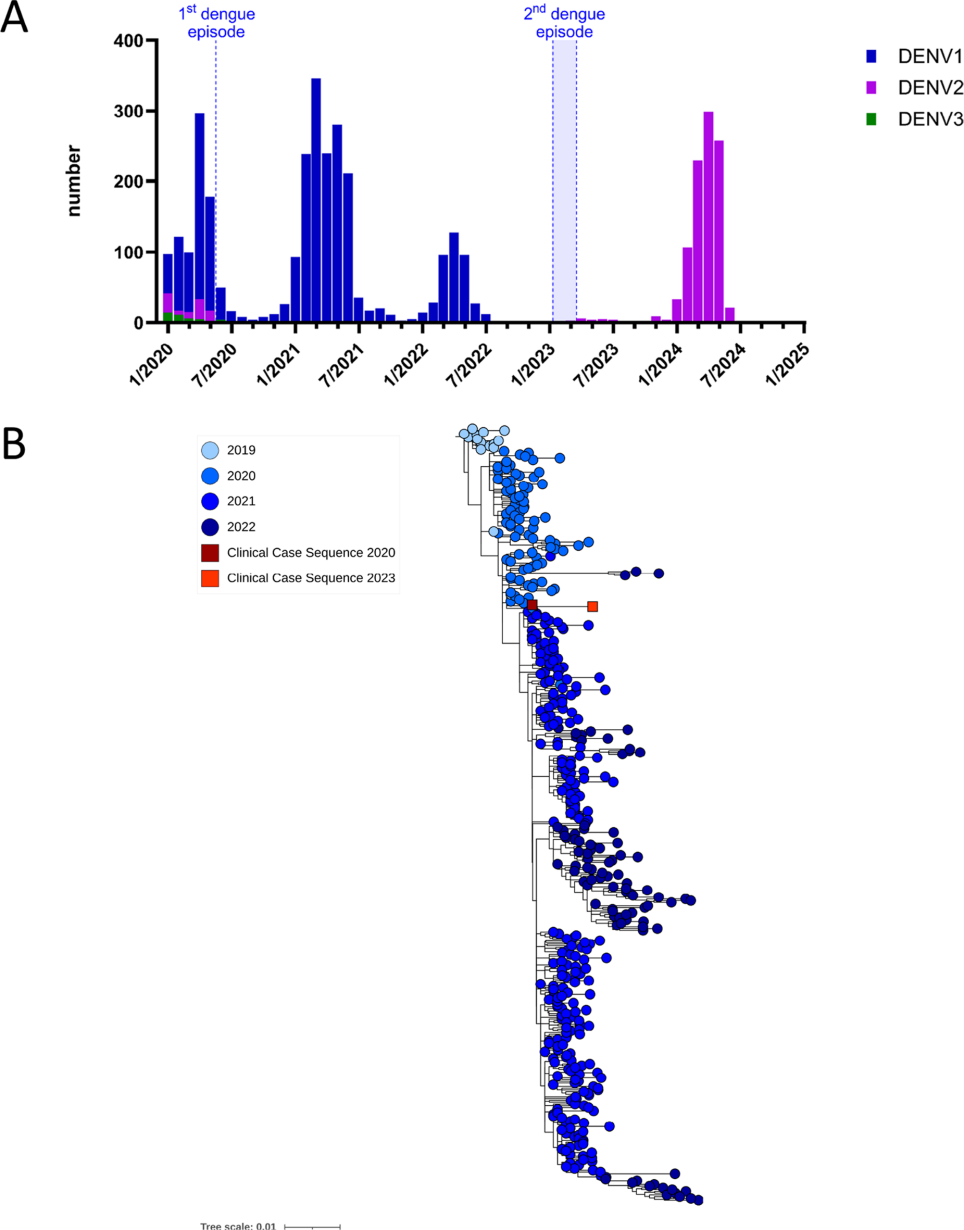
Comparison of DENV sequences from this clinical case. **A** Summary of DENV serotypes detected on Reunion Island from 2020 to 2024 (as of July 2024) as determined by RT-PCR. **B** Phylogenetic relationships of DENV-1 genotype I sequences from this case, including a sequence from the sample collected on 18/05/2020 and a sequence from the sample collected on 14/02/2023, which had the best coverage and quality. These sequences were compared with other genotype I sequences from 2019 to 2022 detected on Réunion Island, as previously described by Frumence et al. 2024 [4]. The phylogenetic tree was generated via a maximum likelihood (ML) approach with MAFFT alignment and IQ-TREE software with the best-fitting model and 1,000 ultrafast bootstrap replicates

In May 2020, she presented with her first episode of DENV-1 infection, confirmed by positive RT-PCR in her blood (Fig. 1A). This serotype was the predominant strain circulating in the region at the time of infection (Fig. 2A). She exhibited a mild form of dengue fever, but it was complicated by Evans syndrome, characterized by thrombocytopenia (Fig. 1F) and hemolytic anemia (Fig. 1G), which was confirmed by a positive Coombs test. At that time, she was being treated with MMF. A strong immune response characterized by elevated levels of IgM and IgG was detected 10 days post-infection (Fig. 1B). This prompted the discontinuation of MMF, the administration of immunoglobulin infusions, and injections of rituximab (anti-CD20 monoclonal antibody), along with corticosteroids, which were rapidly tapered and then discontinued. Entecavir was introduced due to a history of past hepatitis B virus (HBV) infection with serological immunity. Her kidney function deteriorated, leading to the initiation of dialysis in July 2020. She subsequently did not receive any further immunosuppressive therapy. Entecavir was discontinued in December 2021. She completed her SARS-CoV-2 vaccination with 4 doses ranging from June 2021 to January 2022 but did not develop an antibody response (Fig. 1C). Despite receiving prophylactic tixagevimab/cilgavimab (EVUSHELD) in April 2022, she tested positive for an asymptomatic SARS-CoV-2 infection in July 2022. Unfortunately, it was not possible to characterize the strain by sequencing. She received a second prophylactic EVUSHELD infusion in October 2022.

In December 2022, the patient was hospitalized for high fever, anorexia, dyspnea, and diarrhea. She was diagnosed with whooping cough, confirmed by a positive PCR for *Bordetella pertussis* on a nasopharyngeal swab, as well as a digestive infection caused by *Aeromonas* spp. Treatment included ciprofloxacin, rovamycin, and ceftriaxone. Soon after, in January 2023, she was diagnosed with a probable nosocomial SARS-CoV-2 infection acquired in her hospital room (Fig. 1A). This infection was characterized by sequencing and was due to the BQ.1.1 sublineage of the Omicron variant, which began circulating in our region at the end of 2022. The diagnosis was marked by a high viral load and mild pulmonary involvement on the CT scan.

She was treated with corticosteroids, which led to a favorable outcome. During that time, routine testing for HBV revealed a loss of antibodies against the virus and low-level DNA detection, prompting the re-initiation of Entecavir. In February 2023, she experienced an episode of choleriform diarrhea without fever, leading to hospitalization. She did not exhibit any respiratory symptoms. A comprehensive infectious workup was conducted, given her immunocompromised status. Surprisingly, RT-PCR for DENV-1 returned positive in the blood, with a high Ct value. No hemorrhagic manifestations or hepatitis were observed. She was thrombocytopenic, with a recent decline in her platelet count from 134 to 77 G/L (Fig. 1F). She presented with inflammatory anemia less than 7 g / dL, and there was no evidence of lupus flare-up or hemolytic anemia (Fig. 1G).

After two days of symptomatic treatment, she was discharged home. Three weeks later, in March 2023, she presented with fever, chills, myalgia, and arthralgia, leading to a new hospitalization. No respiratory or bleeding symptoms were noted. During this hospitalization, she again developed choleriform diarrhea. A thorough infectious evaluation was conducted. RT-PCR test was positive again for DENV-1, and her clinical symptoms were consistent with DENV infection according to WHO criteria. A COVID-19 test was also positive; however, the high Ct value and absence of respiratory symptoms made COVID-19 a less likely cause of the clinical presentation, though it could not be definitively excluded. Partial sequencing of the SARS-CoV-2 virus again revealed the presence of the BQ.1.1 variant, which could suggest either reinfection with the same variant or persistence and incomplete clearance of the virus in this patient (Fig. 1A).

Additionally, a low level of cytomegalovirus (CMV) DNAemia was detected (Fig. 1A), and all other tests were negative. She did not develop any complications from dengue fever, particularly hemorrhagic manifestations or capillary leakage. The patient was severely immunocompromised, with a residual gamma globulin level of 2.7 g/L and a lymphopenia of 0.2 G/L (Fig. 1D and E). She received an infusion of replacement immunoglobulins to achieve a target level of 8 g/L. Her symptoms improved within days, and she was discharged home. Follow-up monitoring revealed complete resolution of symptoms and negative DENV detection one week after discharge.

Retrospective analysis revealed that DENV RNA could be detected in plasma since January 2023. During this period, her level of anti-DENV IgG was below the threshold of positivity. Viral isolations on Vero cells were attempted on all available positive samples, and the virus was successfully isolated from a plasma sample collected on March 3, 2023 (Fig. 1A), demonstrating positive viremia and productive infection.

We performed amplicon-based DENV genome sequencing via Nanopore next-generation sequencing (NGS) technology on all available samples and the isolated virus. All sequences belonged to genotype I of DENV-1 and were very similar after alignment. The February 14, 2023 sample had the most complete sequence with greater depth and quality. The 2020 and 2023 sequences were 99.86% identical, with 12 SNPs resulting in 6 different amino acids. A comparison with DENV-1 genotype I sequences circulating in the region from 2019 to 2022 via ML tree construction revealed that the two sequences clustered within the same clade (bootstrap value of 100%) and most likely derive from each other (Fig. 2B). Although the sequence from the isolated virus also had high coverage and was very similar, it included additional mutations possibly acquired during passage through Vero cells.

In 2023, 50 dengue cases were confirmed on Reunion Island by RT-PCR [8], of which 30 cases (60%) underwent serotyping (Fig. 2A). DENV-2 was the sole serotype detected throughout 2023, and the last recorded detection of DENV-1 on the island was in July 2022. As the patient did not travel outside the territory, reinfection with a different DENV-1 strain is considered highly unlikely. This genomic analysis strongly supports the possibility that persistent DENV infection is reactivated rather than homotypic reinfection. Altogether, these findings illustrate a rare case of prolonged DENV-1 infection, demonstrated by the persistent detection of both viral RNA and infectious virions from the same strain long after the acute phase in an immunocompromised patient.

## Discussion and conclusions

Here, we report a case of possible chronic dengue infection in a dialysis patient with an immunocompromised status who presented symptomatic reactivation three years after the initial infection. Genomic analysis confirmed that the same strain of DENV-1 was present in both episodes, challenging the traditional view of dengue as an acute, self-limiting infection.

DNA viruses such as herpesvirus or HBV are well known for their ability to establish chronic infections, either by entering a state of latency or by engaging in continuous replication. As for RNA viruses, they typically cause acute infections that are cleared from the host within a few days, although a small number of them are capable of causing chronic infections. Some viruses, such as the hepatitis C virus, can achieve chronicity that may be either symptomatic or asymptomatic by evading the immune system through high mutation rates, residing in immunologically privileged sites, and continuously generating infectious virions [9]. SARS-CoV-2 may persist in the respiratory tract and intestines, potentially contributing to long-term conditions such as long COVID [10]. In the case of *alphaviruses*, such as the Chikungunya virus, viral persistence has been observed in the joints, with the potential to induce chronic pain for years [11]. Among *flaviviruses*, it has been reported that Zika, West Nile, and tick-borne encephalitis viruses can persist for several months or even years after acute infection in specific tissues or organs, such as the kidneys, brain, or testes [12–14].

Persistence has also been observed in cases of DENV infection. A recent report on a traveler who contracted DENV-2 demonstrated that viral RNA isolated from the red blood cell fraction remained detectable for 89 days, suggesting that circulating blood cells may harbor the virus [15]. Additionally, a case of chronic DENV infection was reported in an immunocompromised patient within the central nervous system several months after possible primary infection, indicating that the brain is a potential site of persistence [16]. The kidney may also harbor DENV, as studies in kidney transplant recipients detected viral RNA in urine months to years post-infection [7, 17]. In our patient, it was not possible to determine the organ or cells that allowed the virus to persist over the years, nor the mechanisms (persistence of nucleic acid, continuous replication, or latency) that facilitated this. Since RNA viruses (except for retroviruses) are not known to establish latency due to their inability to integrate into the human genome, their susceptibility to rapid degradation, and the absence of an episomal form [9], it may be speculated that persistent DENV infection was localized and active, even at low levels. This is readily apparent since our patient's infection was not systemic, and we were unable to detect the virus in the blood by RT-PCR between the two episodes of positivity.

The initial dengue episode occurred after prolonged immunosuppressive therapy for lupus nephritis and shortly after rituximab administration. The administration of rituximab too early after infection may prevent the formation of plasma cells, which play a crucial role in antiviral humoral protection [18]. In the case of COVID-19, exposure to rituximab may profoundly affect B-cell functions involved in anti-SARS-CoV-2 immunity, significantly influencing the clinical and serological course of SARS-CoV-2 infection, as well as long-term immunity [19]. Therefore, it could be proposed that the compromised immune system associated with rituximab treatment in our patient may have contributed to the persistence of the DENV.

Nearly three years after the primary infection, we detected the same strain of DENV, confirmed by genomic analysis, in our symptomatic patient, which we believe represents a relapse of this persistent DENV infection. During this reactivation phase in the blood, the Ct values were consistently very high, ranging between 32.5 and 34.5, close to our detection limit. Although the patient exhibited symptoms compatible with dengue, given the very low viral loads detected, it is possible that these symptoms were due to another infection, such as SARS-CoV-2 or CMV. However, the new onset of worsening symptoms two months after the detection of COVID-19 and the successful isolation of an infectious DENV point toward dengue as the most likely cause.

Immune monitoring in the months leading to reactivation revealed a progressive decline in the total lymphocyte count, starting in November 2022 and peaking in January 2023 when DENV RNA was first detected. Additionally, the patient failed to mount an antibody response following COVID-19 vaccination and showed a loss of previously acquired antibodies against HBV and DENV at the time of DENV reactivation, likely reflecting reduced antibody production by B cells and plasma cells. Furthermore, our patient exhibited prolonged and severe hypogammaglobulinemia for several months prior to DENV reactivation. These immune deficiencies may have been favored by rituximab treatment [20, 21] along with her long history of past chemotherapy and end-stage chronic kidney disease (ESCKD). Patients with ESCKD have impaired host defenses against infections, poor vaccine responses, and defects in innate, humoral, and cellular immunity [22]. Additionally, lymphopenia and hypogammaglobulinemia are conditions associated with an increased risk of opportunistic infections [23, 24]. Furthermore, T-cell immunity, particularly involving CD8 T cells, is believed to be critical for achieving sterilizing immunity against DENV and controlling viral replication [7, 17, 23]. T lymphocytes play a crucial role in managing the replication of latent viruses such as CMV and HBV and in preventing infection [25]. The decline in DENV antibodies and cellular immunity, combined with the presence of hypogammaglobulinemia, may have facilitated the reactivation of DENV infection. Also, our report lacked a comprehensive evaluation of immune parameters, including monitoring of innate immunity and DENV-specific T cells. As a result, we were unable to fully delineate the specific contributions of humoral and cellular immune responses in mediating viral replication and clearance. Nonetheless, it is noteworthy that our patient no longer exhibited subsequent positivity for DENV or SARS-CoV-2 by RT-PCR and experienced complete resolution of her symptoms by the time she received immunoglobulin replacement therapy at levels exceeding 8 g/L, along with a concomitant increase in lymphocyte count above 1 G/L.

Finally, immunodepression during December hospitalization could have been triggered by *Bordetella pertussis*, *Aeromonas* spp., SARS-CoV-2, and corticosteroid use. Indeed, studies have shown that sepsis is a well-known trigger for the reactivation of multiple latent viruses, such as herpesvirus [26]. COVID-19 can impact immune status, with immune suppression observed during the early stages of the disease, and is known to frequently trigger CMV reactivation [27, 28]. Additionally, corticosteroids exert various effects on immune cell activity and inflammatory cytokines [29]. Thus, we cannot exclude the possibility that these infections and corticosteroids may have contributed to both DENV reactivation and CMV reactivation in our patient, given the close timing of the initial hospitalization for whooping cough, COVID-19, and the clinical reactivation of dengue.

While this case provides valuable insights, it is not without limitations. First, the lack of detailed immune phenotyping restricted our ability to fully characterize the patient’s immune status and to understand the immunological mechanisms contributing to viral persistence and reactivation. Second, the small number of time points with DENV testing limited our capacity to comprehensively assess viral dynamics over time. This was largely due to the retrospective nature of the study and the reliance on remaining sample aliquots originally collected for other purposes. Additionally, no DENV testing was performed between the two reactivation episodes, despite the patient being regularly followed at the hospital, as she remained asymptomatic during that period. Third, we were unable to identify the anatomical reservoir that allowed the virus to persist, as this was a retrospective study not involving human research protocols allowing further sampling. These constraints highlight the challenges of studying viral persistence in such settings and underscore the need for prospective studies in immunocompromised patients living in dengue-endemic areas, with longitudinal sampling and comprehensive immune profiling to better understand dengue chronicity.

In conclusion, this case report underscores several critical aspects of DENV infection, particularly the potential for chronic infection and possible symptomatic reactivation in immunocompromised individuals. Although specific immunological and virological factors contributing to chronic DENV infection have not been identified, the patient’s profound immunosuppressive state likely impaired dengue clearance. Additionally, chronic forms of infection may act as reservoirs for the reintroduction of the same dengue virus strain, contributing to its re-emergence and potentially triggering new outbreaks. In vulnerable patients, chronic dengue infection may also contribute to tissue injury, raising further concerns for clinical outcomes. While the generalizability of these findings to other immunocompromised states and immunocompetent individuals remains uncertain, further research is essential to elucidate the mechanisms underlying dengue chronicity, particularly in relation to viral immune evasion and interactions with the immune system.

## Supplementary Information

Supplementary file 1. This study follows the CARE guidelines for case report reporting. A completed CARE checklist has been provided as a supplementary file. In addition, detailed descriptions of the virology and sequencing methods used in this study are included in the supplementary material.

## Data Availability

The consensus sequence from the sample collected on 18/05/2020 and the sequence from the sample collected on 14/02/2023 are available on GenBank under accession numbers PQ732816 and PQ732815.
