# Supplementary material for: Chronic and reactivated dengue infection in an immunocompromised host: insights from a case report
Source: Trop Med Health. 2025 Aug 14;53:107. doi: 10.1186/s41182-025-00779-5 (PMC12351822; doi:10.1186/s41182-025-00779-5)
Supplement: Supplementary file 1 — This study follows the CARE guidelines for case report reporting. A completed CARE checklist has been provided as a supplementary file. In addition, detailed descriptions of the virology and sequencing methods used in this study are included in the supplementary material. [file 41182_2025_779_MOESM1_ESM.docx]

**Methods**

#### *Dengue RT-PCR*

Viral RNA was extracted from 200 µl of the patient’s serum or plasma, or 200 µl of clinical isolates, using the NucliSENS easyMAG kit on the easyMAG or eMAG automation systems (bioMerieux), following the generic extraction protocol as per the manufacturer’s instructions. DENV detection was performed using an in-house one-step quantitative RT-PCR on the Roche LightCycler 480 Real-Time PCR System (Roche), utilizing the TaqMan Fast Virus 1-Step Master Mix for qPCR (Applied Biosystems) assay as described in [1]. Ct values <35 were interpreted as positive. DENV-1 serotyping was performed using the SuperScript III Platinum One-Step Quantitative RT-PCR System (Invitrogen) as described in [2].

#### *Virus Isolation*

#### Vero cell line is derived from kidney tissue of a normal adult African green monkey. The cell line was kindly provided by Pr. Philippe Gasque and cultured in MEM Eagle (PAN BIOTECH) supplemented with 10% heat-inactivated fetal bovine serum (PAN BIOTECH) at 37°C. Virus isolation in a BSL3 facility was performed by inoculating patient serum and plasma (1:10, 1:100, and 1:1000 dilutions) onto Vero cells after two 7-day passages. The supernatant from the cells showing cytopathic effects was frozen and tested by RT-PCR and sequenced to confirm the infection.

#### *DENV Genome Sequencing*

Genome sequencing of DENV was performed directly on viral RNA from positive samples using the amplicon-tiling protocol for the Oxford Nanopore Technologies (ONT) sequencing platform, as described in [3].

### *Phylogenetic analysis*

### Nearly complete genomes were aligned using Geneious Prime software version 2023.1.2 with the MAFFT multiple aligner version 1.5.0, applying default parameters and trimming free-end gaps. Phylogenetic analysis was conducted using the maximum-likelihood method in IQTree version 1.6.12 with 1,000 ultrafast bootstrap replicates. Other DENV-1 sequences from Reunion from 2019-2022 were previously generated and are available on GenBank (OR235231-OR235708). Trees were annotated and visualized using iTOL (<http://itol.embl.de>).

***Graphical Representation and Statistical Analysis***

All graphs were generated using GraphPad Prism (version 10). No statistical comparison was made in this study.

**References**

1. Giry C, Roquebert B, Li-Pat-Yuen G, Gasque P, Jaffar-Bandjee M-C. Simultaneous detection of chikungunya virus, dengue virus and human pathogenic Leptospira genomes using a multiplex TaqMan® assay. BMC Microbiol. 2017;17:105.

2. Leparc-Goffart I, Baragatti M, Temmam S, Tuiskunen A, Moureau G, Charrel R, et al. Development and validation of real-time one-step reverse transcription-PCR for the detection and typing of dengue viruses. J Clin Virol. 2009;45:61–6.

3. Frumence E, Wilkinson DA, Klitting R, Vincent M, Mnemosyme N, Grard G, et al. Dynamics of emergence and genetic diversity of dengue virus in Reunion Island from 2012 to 2022. PLoS Negl Trop Dis. 2024;18:e0012184.
